# Cost-effectiveness of folic acid therapy for primary prevention of stroke in patients with hypertension

**DOI:** 10.1186/s12916-022-02601-z

**Published:** 2022-10-25

**Authors:** Tiantian Zhang, Zhuoru Liang, Tengfei Lin, David J. Cohen, Alejandro Arrieta, Xiaobin Wang, Xianhui Qin, Binyan Wang, Yong Huo, Gordon G. Liu, Jie Jiang, Zugui Zhang

**Affiliations:** 1 College of Pharmacy/International Cooperative Laboratory of Traditional Chinese Medicine Modernization and Innovative Drug Development of Ministry of Education (MOE) of China, Jinan University, Guangzhou, China; 2 Guangzhou Huabo Biopharmaceutical Research Institute, Guangzhou, China; 3 Department of Pharmacy, The Third Affiliated Hospital (The Affiliated Luohu Hospital) of Shenzhen University, Shenzhen, China; 4 Guangzhou Institutes of Biomedicine and Health, Chinese Academy of Sciences, Guangzhou, China; 5 Shenzhen Institutes of Advanced Technology, Chinese Academy of Sciences, Shenzhen, China; 6 Cardiovascular Research Foundation, New York, NY USA; 7 St. Francis Hospital, Roslyn, NY (D.J.C) USA; 8 Department of Health Policy and Management, Robert Stempel College of Public Health and Social Work, Florida International University, Miami, FL USA; 9 Department of Population, Family and Reproductive Health, Johns Hopkins University Bloomberg School of Public Health, Baltimore, MD USA; 10 National Clinical Research Study Center for Kidney Disease, The State Key Laboratory for Organ Failure Research, Renal Division, Nanfang Hospital, Southern Medical University, Guangzhou, China; 11 Shenzhen Evergreen Medical Institute, Shenzhen, China; 12 Institute of Biomedicine, Anhui Medical University, Hefei, China; 13 Department of Cardiology, Peking University First Hospital, Beijing, China; 14 National School of Development, Peking University, Beijing, China; 15 Institute for Global Health and Development, Peking University, Beijing, China; 16 Institute for Research in Equity and Community Health, Christiana Care Health System, Newark, DE USA

**Keywords:** Cost-effectiveness, Folic acid, Primary prevention, Stroke, Hypertension

## Abstract

**Background:**

For hypertensive patients without a history of stroke or myocardial infarction (MI), the China Stroke Primary Prevention Trial (CSPPT) demonstrated that treatment with enalapril-folic acid reduced the risk of primary stroke compared with enalapril alone. Whether folic acid therapy is an affordable and beneficial treatment strategy for the primary prevention of stroke in hypertensive patients from the Chinese healthcare sector perspective has not been thoroughly explored.

**Methods:**

We performed a cost-effectiveness analysis alongside the CSPPT, which randomized 20,702 hypertensive patients. A patient-level microsimulation model based on the 4.5-year period of in-trial data was used to estimate costs, life years, quality-adjusted life years (QALYs), and incremental cost-effectiveness ratios (ICERs) for enalapril-folic acid vs. enalapril over a lifetime horizon from the payer perspective.

**Results:**

During the in-trial follow-up period, patients receiving enalapril-folic acid gained an average of 0.016 QALYs related primarily to reductions in stroke, and the incremental cost was $706.03 (4553.92 RMB). Over a lifetime horizon, enalapril-folic acid treatment was projected to increase quality-adjusted life years by 0.06 QALYs or 0.03 life-year relative to enalapril alone at an incremental cost of $1633.84 (10,538.27 RMB), resulting in an ICER for enalapril-folic acid compared with enalapril alone of $26,066.13 (168,126.54 RMB) per QALY gained and $61,770.73 (398,421.21 RMB) per life-year gained, respectively. A probabilistic sensitivity analysis demonstrated that enalapril-folic acid compared with enalapril would be economically attractive in 74.5% of simulations at a threshold of $37,663 (242,9281 RMB) per QALY (3x current Chinese per capita GDP). Several high-risk subgroups had highly favorable ICERs < $12,554 (80,976 RMB) per QALY (1x GDP).

**Conclusions:**

For both in-trial and over a lifetime, it appears that enalapril-folic acid is a clinically and economically attractive medication compared with enalapril alone. Adding folic acid to enalapril may be a cost-effective strategy for the prevention of primary stroke in hypertensive patients from the Chinese health system perspective.

**Supplementary Information:**

The online version contains supplementary material available at 10.1186/s12916-022-02601-z.

## Background

Stroke is the second leading cause of death worldwide and the total number of deaths from stroke reached 6.55 million in 2019 [1]. As 77% of strokes are first events, effective primary prevention for stroke is essential to halt or reverse its rising burden [2]. Previous trials and meta-analyses have indicated that supplementation with folic acid might be an effective therapy for the primary prevention of stroke [3–5]. The recent China Stroke Primary Prevention Trial (CSPPT) enrolled a total of 20,702 patients with hypertension without a history of stroke or myocardial infarction (MI). Patients were randomly assigned in a 1:1 ratio to receive 1 tablet containing 10 mg of enalapril and 0.8 mg of folic acid daily (single-pill compound, the enalapril-folic acid group) or 1 tablet containing 10 mg of enalapril daily (the enalapril group). After a median treatment duration of 4.5 years, the hazard ratio (HR) for occurrence of first stroke comparing enalapril-folic acid versus enalapril was 0.79 (95% CI: 0.68 to 0.93), with a 21% reduction in relative risk of first stroke, demonstrating that enalapril-folic acid was more effective for the primary prevention of stroke compared with enalapril alone [6]. A recent meta-analysis including 22 folic acid trials for the primary prevention of cardiovascular disease (CVD) events among Chinese populations, demonstrated an even greater risk reduction [7].

As one of the most common causes of long-term disability, stroke has a substantial impact on the total cost of healthcare worldwide, especially in developing countries [8, 9]. Although the benefits of folic acid treatment for hypertensive patients are evident, it is unknown whether folic acid treatment provides these health benefits at an acceptable cost to society. Given the large population of patients who might benefit from such therapy, this information is critical for physicians, patients, and policy holders to make informed decisions regarding preventive therapies. To address this gap in knowledge, we used data from the CSPPT to perform a formal health assessment of the cost-effectiveness of enalapril-folic acid versus enalapril alone for the primary prevention of stroke in hypertensive patients.

## Results

### In-trial cost-effectiveness estimation

Table 1 presents the results of the in-trial cost-effectiveness analyses per person. The mean costs of study drugs were $1170.46 (7549.47 RMB) and $137.41 (886.29 RMB) in the enalapril-folic acid and enalapril groups, respectively. The ischemic stroke costs and hemorrhagic stroke costs were both lower for the enalapril-folic acid group, but the costs of CHD events were higher for the enalapril-folic acid group. Total in-trial mean cost remained significantly higher for the enalapril-folic acid group [$923.74 (5958.14 RMB) versus $217.71 (1404.22 RMB) for the enalapril group, difference: $706.03 (4553.92 RMB)]. The QALY was 0.016 higher in the enalapril-folic acid group during the in-trial period, indicating that the addition of folic acid to enalapril offered a benefit in quality-adjusted life-years. In the in-trial period, the ICER of enalapril-folic acid compared with enalapril was $44,127.13 (284,620 RMB) per QALY, which was higher than the WHO recommended willingness-to-pay threshold of 3 times the GDP per capita ($37,663; 242,928 RMB).

**Table 1:** In-trial cost-effectiveness results

Outcomes	Enalapril-folic acid (***n*** = 10348)	Enalapril (***n*** = 10354)	∆ (Enalapril-folic acid–Enalapril) (95%CI)	***P*** value
**Enalapril/enalapril-folic acid cost ($/￥)**	$1170.46 ￥7549.47	$137.41 ￥886.29	$1033.05 (1029.51 to 1036.59) ￥6663.18 (6640.35 to 6686.01)	< 0.001
**Concomitant drug cost ($/￥)**	$75.13 ￥484.60	$73.01 ￥470.91	$2.12 (− 2.20 to 6.44) ￥13.69 (− 14.16 to 41.54)	0.335
**Stroke-related costs ($/￥)**				
Ischemic stroke	$32.95 ￥212.55	$43.13 ￥278 .16	$− 10.17 (− 16.66 to − 3.68) ￥− 65.61 (− 107.45 to − 23.76)	0.002
Hemorrhagic stroke	$17.98 ￥115.96	$19.21 ￥123.88	$− 1.23 (− 7.86 to 5.41) ￥− 7.93 (− 50.72 to 34.87)	0.717
**Other CVD-related costs ($/￥)**	$10.89 ￥70.25	$9.98 ￥64.36	$0.91 (− 5.11 to 6.94) ￥5.89 (− 32.97 to 44.75)	0.766
**Total costs ($/￥)**	$923.74 ￥5958.14	$217.71 ￥1404.22	$706.03 (695.28 to 716.79) ￥4553.92 (4484.56 to 4623.27)	< 0.001
**QALY**	3.92	3.90	0.016 (− 0.001 to 0.033)	0.064
**ICER ($/QALY)/(￥/QALY)**	$44,127.13 ￥284,620			

*QALY* quality-adjusted life-year, *ICER* incremental cost-effectiveness ratio

### Lifetime cost-effectiveness estimation

Table 2 shows the results of the lifetime cost-effectiveness analyses for base case and scenarios using a microsimulation model. Total lifetime cost remained significantly higher (by $1633.84; 10,538.27 RMB) for the enalapril-folic acid group ($3903.69 versus $2269.85). The enalapril-folic acid treatment offered an advantage over the enalapril treatment of 0.06 QALY or 0.03 life-year. The ICER for the enalapril-folic acid treatment, compared to the enalapril treatment, was $26,066.13 (168,126.54 RMB) per QALY gained and $61,770.73 (398,421.21 RMB) per life-year gained, respectively. When the unit price of enalapril-folic acid was reduced by 50% under the PAP scenario, the ICER for lifetime enalapril-folic acid treatment strategies was reduced to $8279.10 (53,400.20 RMB) per QALY. Scenario analyses of the survival benefit of enalapril-folic acid show that, when the survival benefit of enalapril-folic acid was assumed to remain constant through 10 years, the ICER for lifetime enalapril-folic acid treatment was $33,012.65 (212,931.59 RMB) per QALY, less than the threshold of 3 times the GDP per capita; however, when the duration of benefit was limited to 5 years, the ICER for enalapril-folic acid treatment increased to $45,246.92 (291,842.63 RMB) per QALY, which is greater than the WHO-recommended threshold.

**Table 2:** Lifetime cost-effectiveness results for base case and scenario analyses

Outcomes	Cost ($/￥)			Effectiveness (QALY or life-years)			ICER	% < 1 time of GDP/capita per QALY	% < 2 times of GDP/capital per QALY	% < 3 times of GDP/capita per QALY
	Enalapril-folic acid	Enalapril	∆	Enalapril-folic acid	Enalapril	∆				
**Base case analyses**										
Quality-adjusted life-year	$3903.69 ￥25,178.80	$2269.85 ￥14,640.53	$1633.84 ￥10,538.27	11.06	11.00	0.06	$26,066.13 ￥168,126.54	2.6%	47.6%	74.5%
Life-year	$3903.69 ￥25,178.80	$2269.85 ￥14,640.53	$1633.84 ￥10,538.27	22.59	22.56	0.03	$61,770.73 ￥398,421.21	6.4%	27.1%	38.6%
**Scenario analyses**										
Enalapril-folic acid at 1/2 cost	$2788.79 ￥17,987.70	$2269.85 ￥14,640.53	$5018.94 ￥3347.16	11.06	11.00	0.06	$8279.10 ￥53,400.20	73.8%	91.3%	94.7%
Enalapril-folic acid has no benefit beyond year 5	$4034.69 ￥26,023.75	$2251.04 ￥14,519.21	$1783.66 ￥11,504.61	11.045	11.01	0.04	$45,246.92 ￥291,842.63	0	9.4%	32.7%
Enalapril-folic acid has no benefit beyond year 10	$3965.16 ￥25,575.28	$2247.02 ￥14,493.28	$1718.15 ￥11,082.07	11.06	11.01	0.05	$33,012.65 ￥212,931.59	0.1%	29.5%	59.5%

*QALY* quality-adjusted life-year, *ICER* incremental cost-effectiveness ratio

**Fig. 1:**
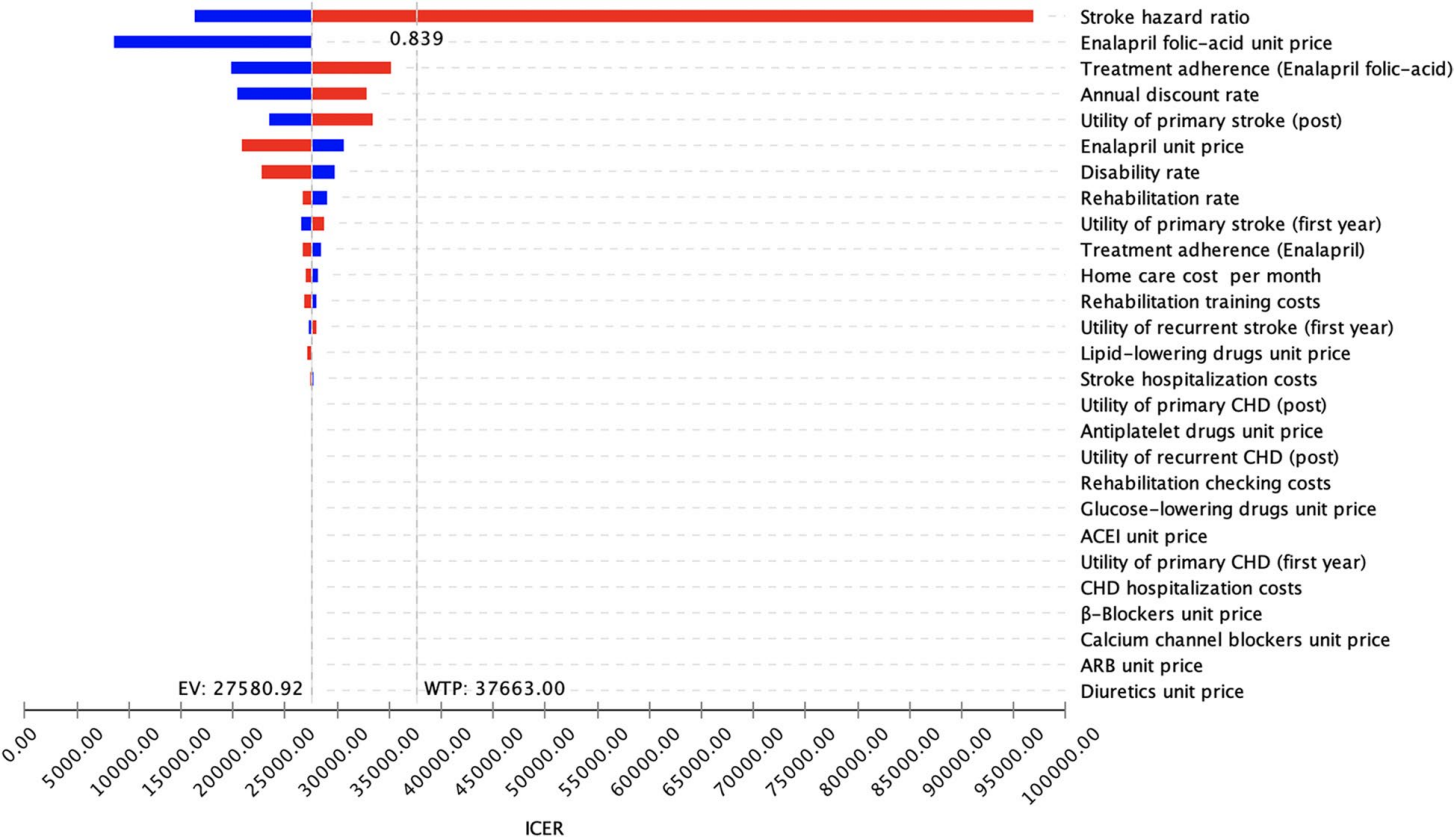
Tornado plot demonstrating the impact of varying each of the model parameters on the ICER for enalapril-folic acid versus enalapril

The results of the one-way sensitivity analyses are summarized in Fig. 1. The model was most sensitive to the HR for stroke with enalapril-folic acid vs. enalapril monotherapy. The ICER for enalapril-folic acid exceeded the WHO-recommended threshold for acceptable cost-effectiveness in China at a HR >0.84, and at the upper bound of the 95% CI for the HR, the ICER for enalapril-folic acid was $96,887.41 (624,923.79 RMB) per QALY gained. Other variables, such as adherence rate of enalapril-folic acid and enalapril treatment, discount rate, unit price of enalapril, and disability rate after stroke, had a moderate or mild impact on the economic outcomes.

**Fig. 2:**
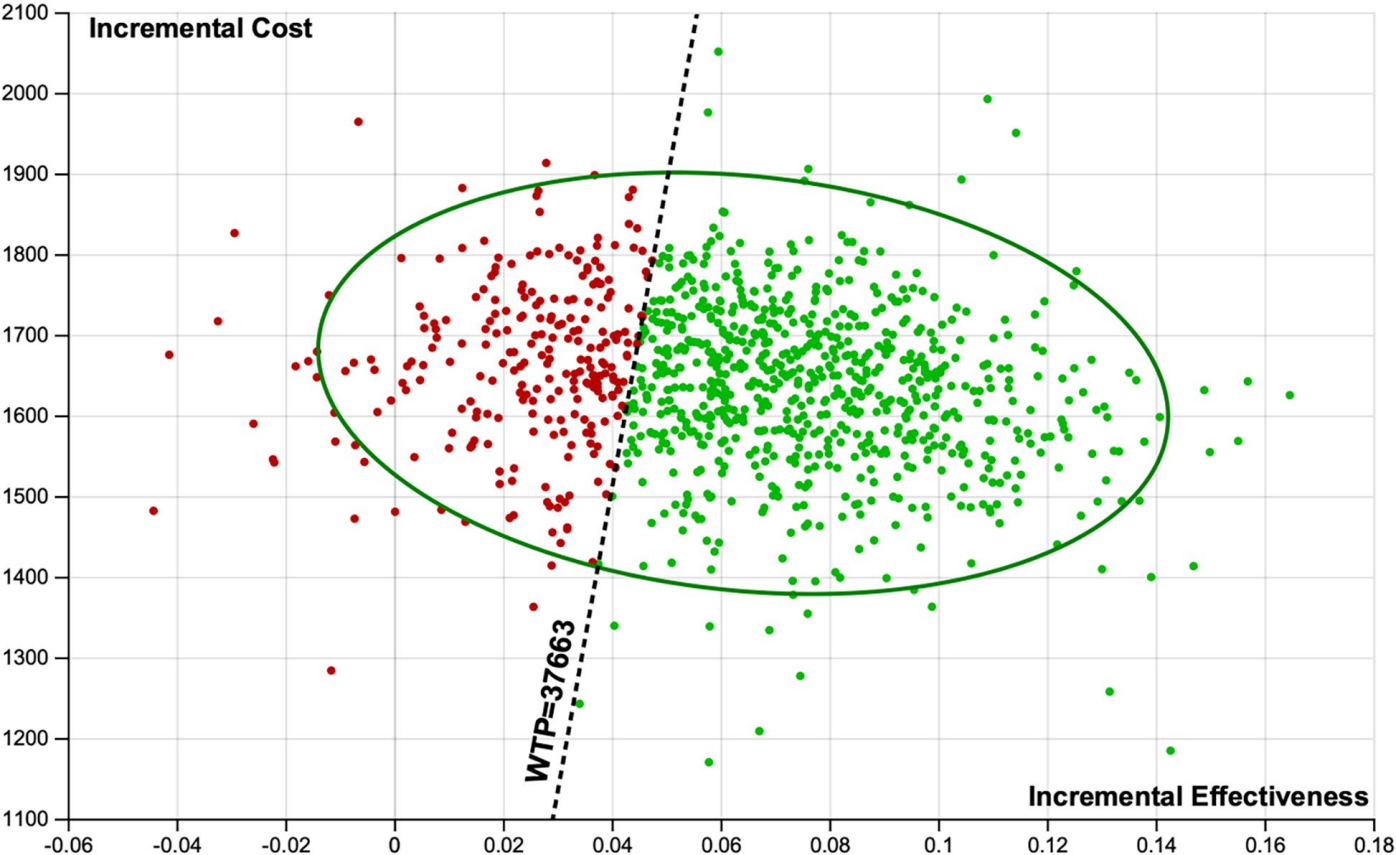
Scatterplot of enalapril-folic acid vs enalapril in the cost-effectiveness plane

**Fig. 3:**
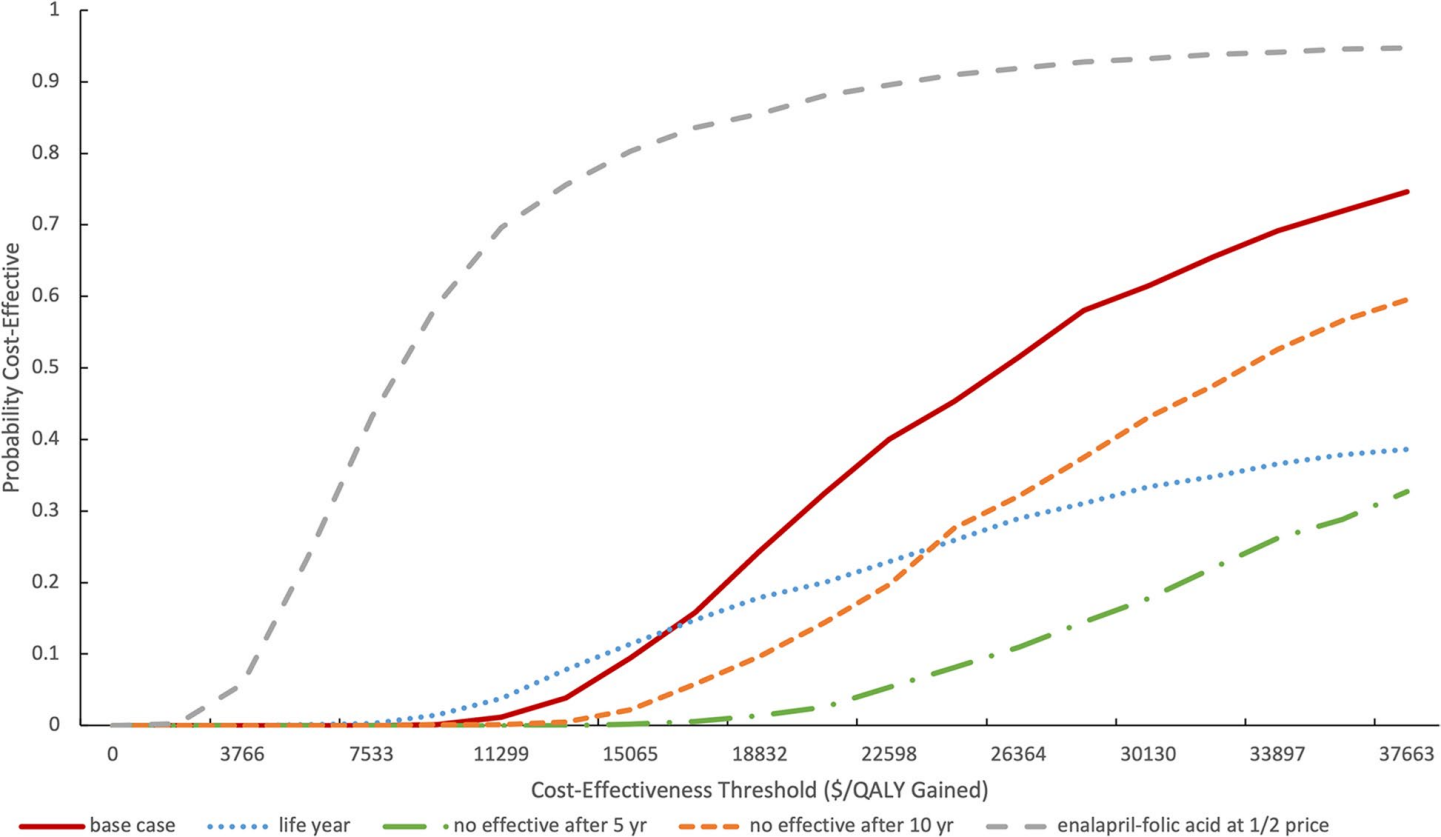
Cost-effectiveness acceptability curve of enalapril-folic acid versus enalapril. Cost-effectiveness acceptability curve of enalapril-folic acid versus enalapril, for the base case (red line), life year (blue), and scenario analyses: no effect of enalapril-folic acid after 5 years (green); no effect of enalapril-folic acid after 10 years (orange); enalapril-folic acid at 1/2 price (gray). QALY, quality-adjusted life-year

Our probabilistic sensitivity analyses demonstrated that the strategy of enalapril-folic acid compared with enalapril was cost-effective in 74.5% of simulations at a WTP threshold of 3 times the GDP per capita ($37,663; 242,928 RMB) (Fig. 2). The impact of alternative assumptions regarding the cost and duration of benefit of enalapril-folic acid are displayed as cost-effectiveness acceptability curves in Fig. 3. When outcomes were assessed under the PAP scenario, the probability that enalapril-folic acid would be cost-effective improved to 94.7%. The cost effectiveness of enalapril-folic acid was also sensitive to the duration of benefit compared with enalapril monotherapy. When we assumed that enalapril-folic acid would continue to incur costs but have no benefit after 10 years, the probability of enalapril-folic acid being cost-effective based on a threshold of 3 times the GDP per capita fell to 59.5%, and when we assumed that enalapril-folic acid would have no effect after 5 years, the probability of its being cost-effective fell to only 32.7%.

### Subgroup analysis

Results from prespecified subgroup analyses are summarized in Additional file 1: Table S1. The ICER of males was $14,091.71 (90,891.71 RMB) per QALY gained and the probability that enalapril-folic acid therapy would be more cost-effective at a threshold ICER of 3 times the GDP per QALY was 92.0%; in contrast to what has been shown in females; however, the ICER was $65,634.34 (423,341.49 RMB) per QALY, which was far beyond the threshold ICER of 3 times the GDP per QALY and the probability at that threshold was only 13.0%. The ICER for people aged 55 to 64 was lower [$15,956.14 (102,917.10 RMB) per QALY], with a 91.1% probability below the threshold ICER of 3 times the GDP per QALY, compared to people younger than 55 years [$64,893.23 (418,561.33 RMB) per QALY] or older than 65 years [$56,003.24 (102,917.10 RMB) per QALY]. In the smoking status subgroup, the cost-effectiveness was more favorable in former smokers [$18,146.57 (117,045.38 RMB) per QALY] compared with current smokers [$24,746.65 (159,615.89 RMB) per QALY]. For people with the MTHFR CT genotype, the probability was 2.6% at a threshold ICER of 3 times the GDP per capita for all patients, while the probabilities of people with the MTHFR CC and TT genotypes being below the threshold were both over 90% at that threshold. Compared to patients with lower risk factor levels, the lifetime ICER was more favorable in patients with higher risk factor levels (diabetes, higher SBP, higher total cholesterol, higher HDL-C, lower folate, and higher homocysteine).

## Discussion

The CSPPT is the first, large-scale, randomized, controlled trial to demonstrate that treatment with enalapril-folic acid resulted in a significant reduction of primary stroke compared with treatment with enalapril alone for patients with hypertension [6, 10]. The results from this prospectively designed health economic analysis carried out alongside the CSPPT revealed that the ICER for enalapril-folic acid versus enalapril was $44,127.13 (284,620.00 RMB) per QALY gained during the 4.5-year follow-up period and $26,066.13 (168,126.54 RMB) per QALY gained over the remaining lifetime. These results were robust to a broad range of sensitivity analyses over a series of alternative assumptions, with an acceptability rate of 74.5% at the WHO-recommended WTP threshold of $37,663 (242,928 RMB) per QALY gained. In terms of the general population, these results suggest that enalapril-folic acid therapy is a cost-effective strategy for primary stroke prevention in hypertensive patients in China.

The subgroup analyses revealed some heterogeneity in the ICER estimates. The results indicated that, lifetime enalapril-folic acid treatment generally yielded ICERs below the “cost-effective” (< 3 times the GDP/capita) threshold for most patients, and some patients were even in the “highly cost-effective” range, such as patients with higher-risk factor levels (diabetes, baseline SBP ≥ 180 mm Hg, total cholesterol ≥ 6.2 mmol/L, or folate < 5.6 ng/mL). In a resource-constrained healthcare environment, targeting therapy to these subgroups of patients would be a highly efficient strategy for maximizing health benefits while minimizing the incremental costs of therapy. On the other hand, there were several subgroups of patients (e.g., female patients, aged < 55 or ≥ 65 years, MTHFR CT genotype, SBP < 160 mm Hg, total cholesterol < 5.2 mmol/L, folate ≥ 10.5 ng/mL, or homocysteine ≦ 10.0 μmol/L), for whom lifetime enalapril-folic acid therapy is “not cost-effective” (ICERs > 3 times the GDP/capita). These results reflect that for younger female patients with the MTHFR CT genotype and lower-risk factor levels, prolonged enalapril-folic acid therapy is not an economical strategy. Since the reduced sample sizes in each subgroup may lead to greater uncertainty, further studies are warranted to confirm the clinical and cost-effectiveness of enalapril-folic acid therapy for patients with different levels of risk factors of stroke at baseline.

### Comparison with previous studies

Many previous studies have evaluated the cost-effectiveness of strategies for the primary prevention of CVD [11–13]. However, few studies have investigated the cost-effectiveness of CVD prevention in China. Xie et al. showed that intensive hypertension control would prevent about 2.2 million CHD events and 4.4 million stroke events for all hypertensive patients in 10 years. Additionally, intensive hypertension treatment has been shown to be more cost-effective than standard hypertension control in China, with an ICER of $1190 per QALY [14]. Basu et al. predicted that the simple benefit-based tailored treatment (BTT) strategy was more effective than the treat-to-target (TTT) or hybrid strategies in reducing CVD mortality, with $142–$182 less per disability-adjusted-life-years gained (DALY) than either the TTT or hybrid strategies [15]. In another study, Gu et al. demonstrated that it is necessary to provide low-cost essential antihypertensive medicines to expand hypertension treatment. Treating all hypertensive patients (primary and secondary prevention) would prevent about 800,000 CVD events annually and is borderline cost-effective compared with treating only CVD and stage two patients ($13,000 per QALY gained in 2015) [16].

These previous studies, as well as guidelines from the American Heart Association (AHA), confirm the cost-effectiveness of traditional strategies for CVD prevention such as the management of blood pressure, lipid levels, and glucose levels, as well as some other well-recognized risk factor controls [17–19]. According to our study, folic acid supplementation, as a novel CVD preventive strategy, has proven to be cost-effective, especially for patients with specific characteristics. In addition, our study has the unique aspect that it does not rely solely on epidemiologic modeling but is based largely on empirical outcomes data from a large, prospective randomized clinical trial.

## Limitations

This study should be interpreted in light of several important limitations. First, our study extrapolated 4.5 years of in-trial data to a lifetime horizon to capture the full benefits of the enalapril folic-acid treatment strategy and assumed that the impact of enalapril folic-acid treatment on the long-term risk of stroke was maintained throughout a patient’s life. However, our scenario analysis results show that even when enalapril-folic acid has no effect after 10 years—an unlikely assumption given the age range of patients in the CSPPT—it would still have an 84% probability of being cost-effective. Second, although microsimulation models have the advantage of simulating the complexity between individual risk factors and disease trajectories compared with the Markov model, they are still limited by the quality of data used to construct these models and their underlying model assumptions [20]. Third, although many of the key inputs were derived directly from the CSPPT, a few parameters (such as annual probabilities of first CHD, the utilities of primary stroke and CHD, mortality rates after stroke and CHD events) were estimated based on external studies or databases, which may have introduced uncertainty into the final results. Therefore, all model inputs were evaluated over a wide range of values in the sensitivity analyses, to examine their influence on the robustness of the model results. Fourth, this cost-effectiveness analysis was based on a single randomized trial with restrictive recruitment and limited follow-up. The results need to be validated in other trials since the clinical event rates may change with time. Finally, our study was carried out in a Chinese population without mandatory folic acid fortification. In addition to China, there are other countries with a high prevalence of low blood folic acid concentrations [21], and populations living in such low-folate regions may also benefit from supplemental folic acid treatment. However, whether our current findings can be extrapolated to populations in other countries, especially those with different ethnicities, and varying genetic and clinical characteristics, requires further research.

## Conclusions

Based on empirical data from the CSPPT, we discovered that enalapril-folic acid therapy is a more cost-effective strategy for primary stroke prevention than enalapril alone among most hypertensive patients in China. Males between the ages of 55 and 65 who have a higher risk of stroke (e.g. diabetes, higher SBP, higher total cholesterol, higher HDL-C, lower folate, and higher homocysteine levels at baseline) drive the outcomes. Some subgroups with a potential lower risk of stroke may not be cost-effective. However, under the Patients Aid Program which offers a significant reduction in drug price, enalapril-folic acid therapy probably would be cost-effective in more patients of more subgroups. Although our findings should be interpreted with caution, as China bears the largest hypertensive population and stroke incidence of any country, these findings can inform health care policy and treatment guidelines. Finally, our study may also serve as a model for other countries with similar economic status and demographic characteristics, especially those whose populations have low folate intake.

## Methods

### Characteristics of the study population

Baseline characteristics of patients in the economic analysis are shown in Supplemental Additional file 1: Table S2. A total of 10,348 and 10,354 patients were randomized to the enalapril-folic acid group or the enalapril group, respectively. The mean age was 60 (SD, 7.5) years and 8497 (41.0%) were male. There were no significant differences in baseline characteristics between the enalapril-folic acid and enalapril groups (all *P* > 0.05).

### Study design

The cost-effectiveness analysis was conducted for the in-trial period and for a lifetime. The in-trial cost-effectiveness analysis was performed using the piggy-back method and included all patients in the CSPPT. Data on survival, CVD events, utilities, and healthcare resource use were collected through the 4.5-year follow-up period for all patients to calculate QALYs and costs for the in-trial period. In the lifetime cost-effectiveness analysis, we constructed a microsimulation model based on the CSPPT population data to compare the lifetime costs, life years, quality-adjusted life years, and cost-effectiveness of enalapril-folic acid versus enalapril. This study was conducted with approval from the Institutional Review Boards of each study site.

### Model overview

The microsimulation model was run at the individual patient level; for each patient, patient characteristics and risk factors were estimated by repeatedly sampling (without replacement) from relevant probability distributions of risk factors and were used to obtain the probability of a health transition during each cycle. Risk factors included age, sex, systolic blood pressure, total cholesterol, high density lipoprotein-cholesterol (HDL-C), the MTHFR C677T polymorphism, folate levels, homocysteine, history of diabetes, and current tobacco smoking.

**Fig. 4:**
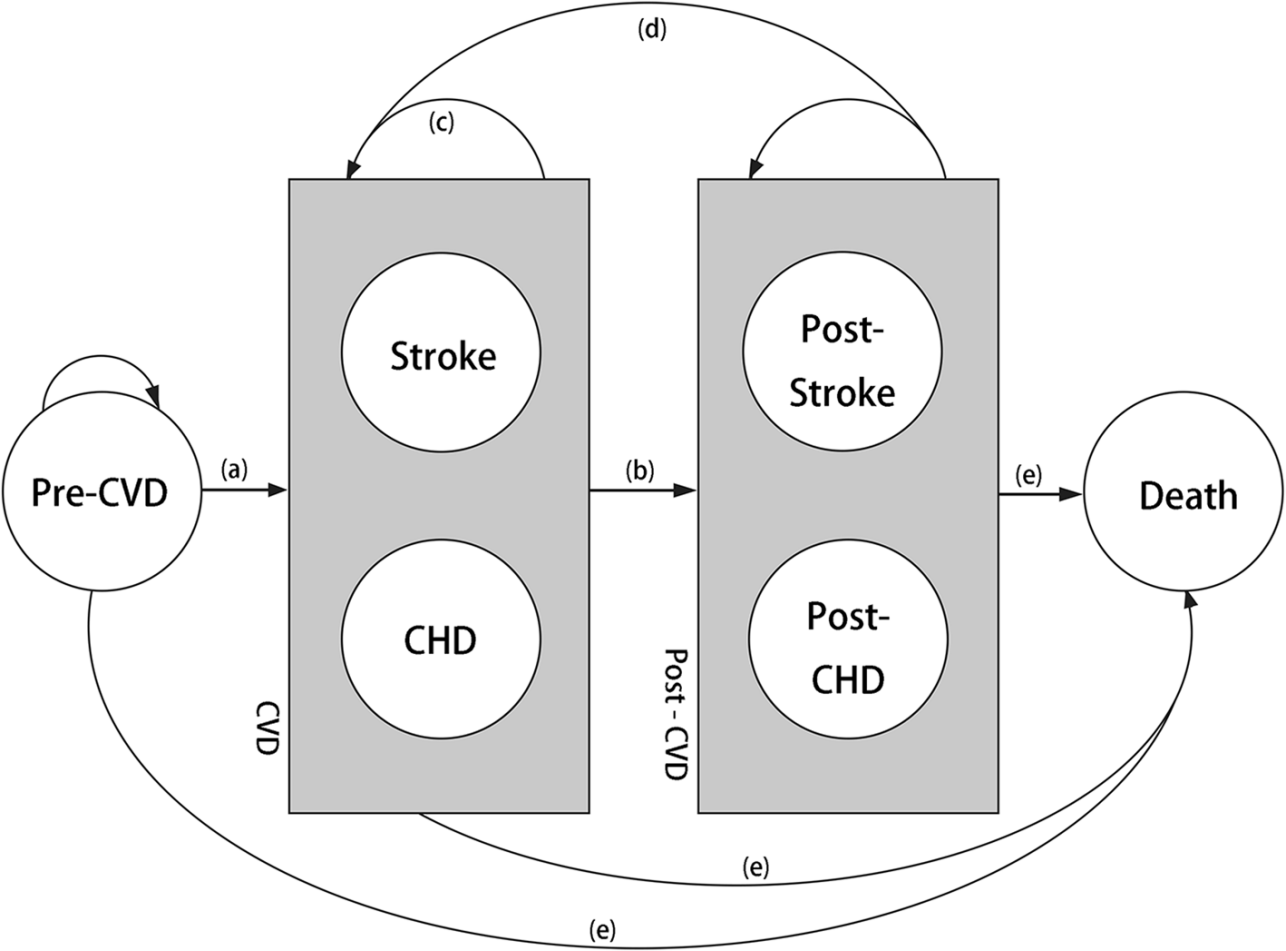
Microsimulation model. **a** 1-year probability of first CVD event. **b** Transition to post-CVD event occurring after one cycle (1 year) with a probability of 100% (tunnel state). **c** CVD relapse during the first year. **d** 1-year probabilities of subsequent CVD events (stroke relapse after first year, post-stroke to CHD, CHD relapse after first year, and post-CHD to stroke). **e** Transition to death (absorbing state). CVD, cardiovascular disease; CHD, coronary heart disease

The structure of our microsimulation model is described in Fig. 4. The model consisted of 6 independent health states: pre-CVD, stroke, coronary heart disease (CHD), post-stroke, post-CHD, and death. In the model, patients with hypertension remained healthy (pre-CVD state) until they developed a stroke (ischemic stroke or hemorrhagic stroke), CHD, or died for any non-CVD-related cause. After the occurrence of a CVD event (including stroke and CHD events in our model), patients were moved to a corresponding post-CVD state, in which they may have a subsequent CVD event or a CVD-related death. The cycle length was set to 1 year. The transition probabilities of our microsimulation model were age-dependent. The parameters of the model are presented in Table 3.

- Annual probability of a first CVD event
  ◦ For the annual probability of a first stroke, we inferred the cycle transition probabilities from the China Stroke Primary Prevention Trial (CSPPT) data [6]. A Weibull distribution was used to model the survival of patients in the enalapril group and to obtain a hypothetical parameters scale (λ) and a shape (γ). The transition probability of enalapril was estimated as follows:
  $$Annual\ probability=1- {\exp}\left[\lambda \ast {(state)}^{\gamma }-\lambda \ast {\left( state+1\right)}^{\gamma}\right]$$
  For enalapril-folic acid, the transition probabilities were estimated by adjusting the hazard ratio (HR=0.79) for enalapril from the CSPPT.
  ◦ Annual probabilities of first CHD were obtained separately for males and females based on methods from the Chinese Multi-provincial Cohort Study (CMCS) [22]. First, the 10-year CHD risk (P) was calculated based on the distribution of risk factors (age, blood pressure, total cholesterol, high-density lipoprotein cholesterol, diabetes, and smoking status) from the CSPPT data. We assumed that a patient’s risk factor (other than age) remained constant from year to year. The 10-year CHD risk was then converted into a 1-year probability of CHD. The transition probability was estimated as follows:
  $${\displaystyle \begin{array}{c}P=1-S{(t)}^{{\exp}\left(f\left[x,M\right]\right)}\\ {}f\left(x,M\right)={\beta}_1\ast \left({x}_1-{M}_1\right)+\dots +{\beta}_p\ast \left({x}_p-{M}_p\right)\\ {} Annual\ probability=1-{\exp}\left(\left({\ln}\left(1-P\right)\right)/10\right)\end{array}}$$
  Because no statistical difference was found in risk of CHD (*p* = 0.89) between the treatment strategies, we assumed no difference in annual probabilities of CHD between the enalapril and the enalapril-folic acid groups.
- Transition to post-CVD event state occurred after one cycle (1 year) with a probability of 100% (tunnel state)
- Annual probabilities of subsequent CVD events (stroke relapse, post-stroke to CHD, CHD relapse, and post-CHD to stroke) were obtained from other studies as primary data from the CSPPT were not available [23–25]
- Transition to death (absorbing state)
  ◦ Age- and sex-specific mortality rates for non-CVD deaths were based on the Chinese life tables obtained from the sixth nationwide census [26]. Because no statistical difference was found in the risk of non-CVD death (*p* = 0.44) between treatment strategies, we assumed no difference in non-CVD mortality rates between the enalapril and the enalapril-folic acid groups.
  ◦ CVD mortality rates at the first year and in subsequent years (after a stroke or after a CHD) were obtained from other studies [23–25]. Because no statistical difference was found in the CVD fatality rate between the enalapril and the enalapril-folic acid groups (*p* > 0.99), we assumed patients had the same chance of dying once they had a stroke.

**Table 3:** Main assumptions for the cost-effectiveness analysis

Parameters	Estimate (SD or range)	Distribution	Reference
**Patient characteristics**			
Age, y	60 (7.53)	Normal	CSPPT [6]
Male sex，%	41%	Table	
**Clinical characteristics**			
Total cholesterol, mg/dL	213.6 (46)	Normal	CSPPT [6]
HDL-C, mg/dL	52 (14)	Normal	
Baseline SBP, mm Hg	139.7 (11.1)	Normal	
Smoking,%	23%	Table	
Diabetes mellitus,%	3%	Table	
**Rates and probabilities**			
Morbidity rates, annual			
Primary stroke	1 − *exp* [*λ* ∗ (*state*)^*γ*^ − *λ* ∗ (*state* + 1)^*γ*^]	Weibull	CSPPT [6]
Primary CHD	1 − *exp* ((*ln*(1 − *P*))/10) *P* = 1 − *S*(*t*)^*exp*(*f*[*x*, *M*])^ *f*(*x*, *M*) = *β*_1_ ∗ (*x*_1_ − *M*_1_) + … + *β*_*p*_ ∗ (*x*_*p*_ − *M*_*p*_)	/	CMCS [22]
Stroke relapse in 1 year	17%	*β*	CKB study [23]
Stroke relapse after 1 year	2–9%	*β*	
Stroke after CHD	0.4%	*β*	The EUROPA study [24]
Stroke after post-CHD	0.4%	*β*	
CHD relapse in 1 year	2.5%	*β*	PEACE [25]
CHD relapse after 1 year	1.2%	*β*	The EUROPA study [24]
CHD after stroke	0.4%	*β*	CKB study [23]
CHD after post-stroke	0.4%	*β*	
Mortality rates, annual			
Non CVD related	1.68–507.28‰ (Age-dependent)	*β*	Life table [26]
After stroke	15%	*β*	CKB study [23]
After post-stroke	3%	*β*	
After CHD	2.8%	*β*	PEACE [25]
After post-CHD	1.5%	*β*	The EUROPA study [24]
**Drug cost per unit, $ (￥)**			
Enalapril	$0.09 ($0.03–$0.22) ￥0.56 (￥0.2–￥1.44)	*γ*	Local bidding Price or the National centralized drug procurement price [27, 28] (see Additional file 1: Table S3)
Enalapril-folic acid	$0.74 ￥4.75	*γ*	
Angiotensin-converting enzyme inhibitors	$0.13 ($0.11–$0.38) ￥0.83 (￥0.72–￥2.43)	*γ*	
Angiotensin II receptor blockers	$0.08 ($0.07–$0.54) ￥0.53 (￥0.48–￥3.48)	*γ*	
Calcium channel blockers	$0.25 ($0.14–$0.47) ￥1.60 (￥0.91–￥3.06)	*γ*	
Diuretics	$0.29 ($0.27–$0.30) ￥1.88 (￥1.72–￥1.95)	*γ*	
β-blockers	$0.03 ($0.02–$0.05) ￥0.21 (￥0.16–￥0.32)	*γ*	
Lipid-lowering drugs	$0.08 ($0.03–$0.65) ￥0.50 (￥0.17–￥4.19)	*γ*	
Glucose-lowering drugs	$0.04 ($0.03–$0.24) ￥0.24 (￥0.19–￥1.57)	*γ*	
Antiplatelet drugs	$0.05 ($0.05–$0.08) ￥0.34 (￥0.31–￥0.50)	*γ*	
**CVD hospitalization costs, $ (￥) (first 30 days)**			
Ischemic stroke	$1529.18 (± 20%) ￥9863.20 (± 20%)	*γ*	China’s Health Statistics Yearbook, 2021 [29]
Hemorrhagic stroke	$3207.55 (± 20%) ￥20,688.70(± 20%)	*γ*	
Coronary heart disease	$4696.39 (± 20%) ￥30,291.70(± 20%)	*γ*	
**Rehabilitation costs per month, $ (￥)**^**a**^			
Rehabilitation training	$200.93 (± 20%) ￥1296.00 (± 20%)	*γ*	National Development and Reform Commission [30] (see Additional file 1: Table S4)
Rehabilitation checking	$7.91 (± 20%) ￥51.00 (± 20%)	*γ*	
Home care	$465.12 (± 20%) ￥3000.00 (± 20%)	*γ*	
**Utility**			
Pre-CVD	0.90 (0.12)	*β*	CSPPT [6]
Primary stroke (first year)	0.76	*β*	Du [31]
Primary stroke (after first year)	0.79	*β*	
Recurrent stroke (first year)	0.30	*β*	Wang [32]
Recurrent stroke (after first year)	0.33	*β*	
Primary CHD (first year)	0.77 (0.75–0.78)	*β*	Goldsmith [33]
Primary CHD (after first year)	0.89 (0.17)	*β*	Wang [34]
Recurrent CHD (first year)	0.64	*β*	Thomas [35]
Recurrent CHD (after first year)	0.76	*β*	
**Treatment adherence**			
Enalaparil-folic acid	0.692 (± 20%)	*β*	CSPPT [6]
Enalapril	0.691 (± 20%)	*β*	
**HR of primary stroke**	0.79 (0.68 -0.93)	*β*	
**Disability rate**	0.39 (0.20–0.8))	*β*	Salomon [8]
**Rehabilitation rate after stroke**	0.58 (0–0.7)	*β*	Asakawa [36]
**Annual discount rates for life years, costs, and QALYs**	5% (0–8%)	/	

^a^Patients with disability after stroke

The model was developed using TreeAge Pro software (TreeAge Software, Inc., Williamstown, MA, USA) (Additional file 2: Fig. S1) and validated by comparing first stroke rates with the 4.5-year in-trial data (Additional file 2: Fig. S2).

### Health care resource use and cost estimation

Medical care costs included pre-CVD drug costs, CVD costs (cost incurred through the first 30 days after a CVD event and for the rest of the first year), and post-CVD costs (cost incurred after the first year) and were assessed from the Chinese healthcare sector perspective. Because the primary endpoint of the CSPPT was first stroke, follow-up was stopped when a stroke occurred. Health care resource use in the in-trial analysis included only pre-stroke drug costs and the first 30 days of hospitalization after CVD events.

Information on pre-CVD medication use and adherence, including the two study drugs (enalapril, enalapril-folic acid) and concomitant medications, was collected for all patients in the CSPPT over a median follow-up period of 4.5 years. Concomitant medications included the five standard antihypertensive drug classes (angiotensin converting enzyme inhibitors, beta blockers, angiotensin II receptor antagonists, long-acting calcium channel blockers, and diuretics), antiplatelet drugs, lipid-lowering drugs, and hypoglycemic drugs. Pre-CVD drug costs were calculated by multiplying the average annual cost of pre-CVD drugs by the number of follow-up years over the in-trial period or the lifetime period and were adjusted by the average treatment adherence time and the percentage of concomitant medication use reported in the CSPPT. The unit price of each medication pill was estimated according to market share values and the National Centralized Drug Procurement (NCDP) prices or the median bidding price of local official documents from 2021 (Additional file 1: Table S3) [27, 28].

The costs of a CVD event during the first 30-days of hospitalization included stroke-related costs (ischemic stroke and hemorrhagic stroke) and CHD-related costs, which were sourced from the China Health Statistics Yearbook 2021 [29]. Treatment procedures for the rest of the first year and the post-CVD phase followed the clinical pathway of rehabilitation for stroke and medication guidance for CHD, respectively [31, 32]. Costs for each treatment procedure were calculated by multiplying the item counts by their respective unit prices, determined by the average prices of the National Development and Reform Commission of the People’s Republic of China in 2021 (Additional file 1: Table S4) [32, 37]. Taking into account that some people may suffer from disability after stroke, we introduced a disability rate and a rehabilitation rate after stroke based on published literature, to adjust for the costs of the rest of the first year and the costs of post-CVD (for cost Estimation see (Additional file 1: Cost estimates for the rest of the first year or in post-CVD phase) [8, 36].

Costs were reported in US dollars and Chinese renminbi (RMB) according to the 2021 exchange rate ($1.00 =6.45 RMB) [30]. All costs were converted to 2021 with the medical care component of the Consumer Price Index (CPI) [38].

### Quality of life estimation

Utility scores [range 0 (equivalent to death) to 1 (equivalent to perfect health)] were obtained from the CSPPT and the medical literature. Utility values of pre-CVD for enalapril or enalapril-folic acid treatment were calculated by using the EuroQOL five-dimension, three-level questionnaire (EQ-5D-3L), which was completed by the CSPPT patients at the exit visit. The calculation formula was based on the Chinese specific EQ-5D-3L value set system [39]. The health state utility scores of stroke (primary and relapse), CHD (primary and relapse), and post-CVD that are presented in Table 3 were derived from published research [31–35]. Quality-adjusted life-years (QALYs) were calculated by multiplying the length of time in a health state by the utility scores associated with that health state.

### Cost-effectiveness outcomes estimation

Cost-effectiveness was expressed as incremental cost-effectiveness ratios (ICERs), which were calculated as the incremental costs divided by the incremental life-years and the incremental QALYs between the two treatments of enalapril-folic acid vs. enalapril. All future costs, life-years, and QALYs were discounted at 5% annually.

The willingness-to-pay (WTP) threshold for cost-effectiveness was set on the WHO-CHOICE-recommended gross domestic product (GDP) per capita-indexed threshold [40]. A treatment strategy was considered “highly cost-effective” if the WTP for cost-effectiveness was less than one time the GDP per capita; “cost-effective” if the WTP was less than three times the GDP per capita; otherwise, the strategy was considered “not cost-effective.” The GDP per capita for China in 2021 was assumed to be $12,544 (80,976 RMB) and the WTP was assumed to be $37,663 (242,928 RMB) [30].

### Subgroup analysis

The hazard ratios for first stroke in pre-specified subgroups (including sex, age, smoking status, diabetes mellitus, systolic blood pressure at baseline, total cholesterol, high density lipoprotein-cholesterol (HDL-C), the MTHFR C677T polymorphism, folate levels and homocysteine) have been published previously. The pre-estimated λ, γ parameters, and hazard ratios (Additional file 1: Table S5) within each subgroup were used for re-calculating the annual probability of first stroke. The relevant distributions of risk factors in each subgroup were refitted based on the CSPPT data and were used to obtain the annual probability of CHD. Other parameters of the model in the subgroup analysis, such as CVD relapse rates, mortality rates, costs, and utilities, were the same as in the base case analysis.

### Sensitivity analysis

We performed extensive sensitivity analyses to assess the robustness of our results to plausible variation in model parameters including utility scores, HRs, and discount rates. One-way sensitivity analyses were performed for each model parameter and displayed as a tornado diagram. The upper and lower bounds of drug costs were derived from the National Centralized Drug Procurement prices from different manufacturers or from bidding prices in different provinces from 2021 [41]. The price range of rehabilitation treatment was derived from prices set by the Development and Reform Commission from various regions in China [42, 43]. The discount rate was set to vary from 0 to 8%. Ranges of other parameters were obtained from reported 95% CIs or by ± 20% of the base-case values if a 95% CI was not available. Probabilistic sensitivity analyses were also performed to evaluate the impact of simultaneous changes in all of the above model parameters by means of Monte Carlo simulation (10,000 replicates). Distributional assumptions for each model parameter are summarized in Table 3.

In China, pharmaceutical companies provide a 50% discount of their original price to eligible patients through the Patients Aid Program (PAP). Therefore, the impact of the PAP was also evaluated in the scenario analyses. Additionally, the base case analysis assumed that the stroke HR for enalapril-folic-acid versus enalapril was fixed throughout the lifetime (HR = 0.79). In scenario analyses, the survival benefit of enalapril-folic acid was assumed to either (a) remain constant for 10 years, with no benefit beyond year 10, or (b) remain constant for 5 years only with no benefit beyond year 5.

## Supplementary Information

**Additional file 1: **Cost estimates for the rest of the first year or in post-CVD phase. **Table S1.** Lifetime cost-effectiveness results for each subgroup. **Table S2.** Baseline characteristics of the study participants. **Table S3.** Unit prices of drugs. **Table S4.** Costs of each treatment procedure. **Table S5.** Hazard ratios and parameters for scale and shape of the subgroup analysis.

**Additional file 2: Figure S1.** Schematic structure of the microsimulation model. **Figure S2.** Model of internal validation. Internal validation of our microsimulation model shows agreement with the 4.5-year period of in-trial data across first stroke rates.

## Data Availability

All data generated or analyzed during this study are included in this published article and its supplementary information files.

## Abbreviations

CHD: Coronary heart disease
CSPPT: China Stroke Primary Prevention Trial
CVD: Cardiovascular disease
EQ-5D-3L: EuroQOL five-dimension, three-level questionnaire
GDP: Gross domestic product
HR: Hazard ratio
ICER: Incremental cost-effectiveness ratio
PAP: Patients Aid Program
QALYs: Quality-adjusted life years
WTP: Willingness-to-pay

## References

[1] Roth GA, Mensah GA, Johnson CO, Addolorato G, Ammirati E, Baddour LM, Barengo NC, Beaton AZ, Benjamin EJ, Benziger CP (2020). Global burden of cardiovascular diseases and risk factors, 1990-2019: update from the GBD 2019 study. J Am Coll Cardiol.

[2] Virani SS, Alonso A, Benjamin EJ, Bittencourt MS, Callaway CW, Carson AP, Chamberlain AM, Chang AR, Cheng S, Delling FN (2020). Heart Disease and Stroke Statistics-2020 update: a report from the American Heart Association. Circulation.

[3] Galan P, Kesse-Guyot E, Czernichow S, Briancon S, Blacher J, Hercberg S, Group SFOC (2010). Effects of B vitamins and omega 3 fatty acids on cardiovascular diseases: a randomised placebo controlled trial. BMJ.

[4] Lonn E, Yusuf S, Arnold MJ, Sheridan P, Pogue J, Micks M, McQueen MJ, Probstfield J, Fodor G, Held C (2006). Homocysteine lowering with folic acid and B vitamins in vascular disease. N Engl J Med.

[5] Wang X, Qin X, Demirtas H, Li J, Mao G, Huo Y, Sun N, Liu L, Xu X (2007). Efficacy of folic acid supplementation in stroke prevention: a meta-analysis. Lancet.

[6] Huo Y, Li J, Qin X, Huang Y, Wang X, Gottesman RF, Tang G, Wang B, Chen D, He M (2015). Efficacy of folic acid therapy in primary prevention of stroke among adults with hypertension in China: the CSPPT randomized clinical trial. JAMA.

[7] Wang WW, Wang XS, Zhang ZR, He JC, Xie CL (2017). A meta-analysis of folic acid in combination with anti-hypertension drugs in patients with hypertension and hyperhomocysteinemia. Front Pharmacol.

[8] Salomon JA, Vos T, Hogan DR, Gagnon M, Naghavi M, Mokdad A, Begum N, Shah R, Karyana M, Kosen S (2012). Common values in assessing health outcomes from disease and injury: disability weights measurement study for the Global Burden of Disease Study 2010. Lancet.

[9] Liu M, Wu B, Wang WZ, Lee LM, Zhang SH, Kong LZ (2007). Stroke in China: epidemiology, prevention, and management strategies. Lancet Neurol.

[10] Zhang T, Lin T, Wang Y, Wang B, Qin X, Xie F, Cui Y, Huo Y, Wang X, Zhang Z, et al. Estimated Stroke-Free Survival of Folic Acid Therapy for Hypertensive Adults: Projection Based on the CSPPT. Hypertension. 2020;75(2):339-46. 10.1161/HYPERTENSIONAHA.119.14102 PMC8020900 31865785

[11] Aarnio E, Korhonen MJ, Huupponen R, Martikainen J (2015). Cost-effectiveness of statin treatment for primary prevention in conditions of real-world adherence--estimates from the Finnish prescription register. Atherosclerosis.

[12] Lazar LD, Pletcher MJ, Coxson PG, Bibbins-Domingo K, Goldman L (2011). Cost-effectiveness of statin therapy for primary prevention in a low-cost statin era. Circulation.

[13] Lin L, Teng M, Zhao YJ, Khoo AL, Seet RC, Yong QW, Yeo TC, Lim BP (2015). Long-term cost-effectiveness of statin treatment for primary prevention of cardiovascular disease in the elderly. Cardiovasc Drugs Ther.

[14] Xie X, He T, Kang J, Siscovick DS, Li Y, Pagan JA (2018). Cost-effectiveness analysis of intensive hypertension control in China. Prev Med.

[15] Basu S, Yudkin JS, Sussman JB, Millett C, Hayward RA (2016). Alternative strategies to achieve cardiovascular mortality goals in China and India: a microsimulation of target- versus risk-based blood pressure treatment. Circulation.

[16] Gu D, He J, Coxson PG, Rasmussen PW, Huang C, Thanataveerat A, Tzong KY, Xiong J, Wang M, Zhao D (2015). The cost-effectiveness of low-cost essential antihypertensive medicines for hypertension control in China: a modelling study. PLoS Med.

[17] Whelton PK, Carey RM, Aronow WS, Casey DE, Collins KJ, Dennison Himmelfarb C, DePalma SM, Gidding S, Jamerson KA, Jones DW (2018). 2017 ACC/AHA/AAPA/ABC/ACPM/AGS/APhA/ASH/ASPC/NMA/PCNA guideline for the prevention, detection, evaluation, and management of high blood pressure in adults: a report of the American College of Cardiology/American Heart Association Task Force on clinical practice guidelines. Hypertension.

[18] Moran A, Gu D, Zhao D, Coxson P, Wang YC, Chen CS, Liu J, Cheng J, Bibbins-Domingo K, Shen YM (2010). Future cardiovascular disease in china: markov model and risk factor scenario projections from the coronary heart disease policy model-china. Circ Cardiovasc Qual Outcomes.

[19] Yang W, Gage H, Jackson D, Raats M (2018). The effectiveness and cost-effectiveness of plant sterol or stanol-enriched functional foods as a primary prevention strategy for people with cardiovascular disease risk in England: a modeling study. Eur J Health Econ.

[20] Krijkamp EM, Alarid-Escudero F, Enns EA, Jalal HJ, Hunink MGM, Pechlivanoglou P (2018). Microsimulation modeling for health decision sciences using R: a tutorial. Med Decis Making.

[21] McLean E, de Benoist B, Allen LH (2008). Review of the magnitude of folate and vitamin B12 deficiencies worldwide. Food Nutr Bull.

[22] Liu J, Hong Y, D’Agostino RB, Wu Z, Wang W, Sun J, Wilson PW, Kannel WB, Zhao D (2004). Predictive value for the Chinese population of the Framingham CHD risk assessment tool compared with the Chinese Multi-Provincial Cohort Study. JAMA.

[23] Chen Y, Wright N, Guo Y, Turnbull I, Kartsonaki C, Yang L, Bian Z, Pei P, Pan D, Zhang Y (2020). Mortality and recurrent vascular events after first incident stroke: a 9-year community-based study of 0.5 million Chinese adults. Lancet Glob Health.

[24] Fox KM, Investigators EUtOrocewPiscAd (2003). Efficacy of perindopril in reduction of cardiovascular events among patients with stable coronary artery disease: randomised, double-blind, placebo-controlled, multicentre trial (the EUROPA study). Lancet.

[25] Song J, Murugiah K, Hu S, Gao Y, Li X, Krumholz HM, Zheng X, China PCG (2020). Incidence, predictors, and prognostic impact of recurrent acute myocardial infarction in China. Heart.

[26] Tabulation of the 2010 population census of the people’s republic of China. http://www.stats.gov.cn/tjsj/pcsj/rkpc/6rp/indexch.htm. Accessed 18 Aug 2022.

[27] The national centralized drug procurement prices. http://www.gzplan.gov.cn/gzplan/index.shtml. Accessed 18 Aug 2022.

[28] Local bidding price for drugs. https://db.yaozh.com/yaopinzhongbiao. Accessed 18 Aug 2022.

[29] National Health Commission. China’s health statistics yearbook 2021. Beijing: The Ministry of Health of the People’s Republic of China; 2021.

[30] National Bureau of Statistics. Statistical bulletin of national economic and social development in 2021. National Bureau of Statistics; 2022.

[31] Du XD, Zhu P, Li ME, Wang J, Meng HD, Zhu CR (2018). Health utility of patients with stroke measured by EQ-5D and SF-6D. J Sichuan Univ (Med Sci Edi).

[32] Wang YL, Pan YS, Zhao XQ, Wang D, Johnston SC, Liu LP, Meng X, Wang AX, Wang CX, Wang YJ (2014). Recurrent stroke was associated with poor quality of life in patients with transient ischemic attack or minor stroke: finding from the CHANCE trial. CNS Neurosci Ther.

[33] Goldsmith KA, Dyer MT, Schofield PM, Buxton MJ, Sharples LD (2009). Relationship between the EQ-5D index and measures of clinical outcomes in selected studies of cardiovascular interventions. Health Qual Life Outcomes.

[34] Wang L, Wu YQ, Tang X, Li N, He L, Cao Y, Chen DF, Hu YH (2015). Profile and correlates of health-related quality of life in Chinese patients with coronary heart disease. Chin Med J (Engl).

[35] Sehested TSG, Bjerre J, Ku S, Chang A, Jahansouz A, Owens DK, Hlatky MA, Goldhaber-Fiebert JD (2019). Cost-effectiveness of canakinumab for prevention of recurrent cardiovascular events. JAMA Cardiol.

[36] Asakawa T, Zong L, Wang L, Xia Y, Namba H (2017). Unmet challenges for rehabilitation after stroke in China. Lancet.

[37] Lee H, Cho J, Shin DW, Lee SP, Hwang SS, Oh J, Yang HK, Hwang SH, Son KY, Chun SH (2015). Association of cardiovascular health screening with mortality, clinical outcomes, and health care cost: a nationwide cohort study. Prev Med.

[38] Consumer price index: medical care. https://fred.stlouisfed.org/series/CPIMEDSL. Accessed 18 Aug 2022.

[39] Liu GG, Wu H, Li M, Gao C, Luo N (2014). Chinese time trade-off values for EQ-5D health states. Value Health.

[40] Griffiths M, Maruszczak M, Kusel J (2015). The who-choice cost-effectiveness threshold: a country-level analysis of changes over time. Value Health.

[41] Kearney PM, Whelton M, Reynolds K, Muntner P, Whelton PK, He J (2005). Global burden of hypertension: analysis of worldwide data. Lancet.

[42] Price of medical service in Yunnan province. http://www.yndpc.yn.gov.cn. Accessed 18 Aug 2022.

[43] Price of medical service in Guangdong province. http://www.gzplan.gov.cn/gzplan/index.shtml. Accessed 18 Aug 2022.

