# Supplementary material for: Cost-effectiveness of folic acid therapy for primary prevention of stroke in patients with hypertension
Source: BMC Med. 2022 Oct 25;20:407. doi: 10.1186/s12916-022-02601-z (PMC9594871; doi:10.1186/s12916-022-02601-z)
Supplement: Supplementary file 1 — Additional file 1: Cost estimates for the rest of the first year or in post-CVD phase. Table S1. Lifetime cost-effectiveness results for each subgroup. Table S2. Baseline characteristics of the study participants. Table S3. Unit prices of drugs. Table S4. Costs of each treatment procedure. Table S5. Hazard ratios and parameters for scale and shape of the subgroup analysis. [file 12916_2022_2601_MOESM1_ESM.docx]

**Cost-Effectiveness of Folic Acid** **Therapy for Primary Prevention of Stroke in Patients with Hypertension**

**Additional file 1**

**Cost estimates for the rest of the first year or in post-CVD phase**

Some people may suffer from disability after stroke and some of these people will receive rehabilitation treatment. Therefore, we introduced a disability rate (0.39)^[1]^ and a rehabilitation rate after stroke (0.58)^[2]^ taken from published literature to adjust the costs of the rest of the first year and the costs of post-CVD.

$$\boldsymbol{\theta}\mathbf{=}\boldsymbol{C}_{\boldsymbol{1}}\boldsymbol{+}\boldsymbol{C}_{\boldsymbol{2}}\boldsymbol{+}\boldsymbol{C}_{\boldsymbol{3}}\boldsymbol{+}\boldsymbol{C}_{\boldsymbol{4}}$$

$$\mathbf{A}\mathbf{=}\boldsymbol{C}_{\boldsymbol{1}}$$

$$\mathbf{W}\mathbf{=}\boldsymbol{\theta}\mathbf{*}\boldsymbol{R}_{\boldsymbol{1}}\boldsymbol{*}\boldsymbol{R}_{\boldsymbol{2}}\boldsymbol{+}\boldsymbol{A}\boldsymbol{*}\left( \boldsymbol{1}\boldsymbol{-}\boldsymbol{R}_{\boldsymbol{1}}\boldsymbol{-}\boldsymbol{R}_{\boldsymbol{2}} \right)$$

θ: Cost of patients with disability after stroke;

A: Cost of patients without disability after stroke;

W: Cost of the rest of the first year or in post-CVD phase;

C_1_: Drug costs per year;

C_2_: Rehabilitation training costs per year;

C_3_: Rehabilitation checking costs per year;

C_4_: Home care costs per year;

R_1_: Disability rate (0.39);

R_2_: Rehabilitation rate after stroke (0.58)

**References**

1. Salomon JA, Vos T, Hogan DR, Gagnon M, Naghavi M, Mokdad A, Begum N, Shah R, Karyana M, Kosen S *et al*: Common values in assessing health outcomes from disease and injury: disability weights measurement study for the Global Burden of Disease Study 2010. *Lancet* 2012, 380(9859):2129-2143.

2. Asakawa T, Zong L, Wang L, Xia Y, Namba H: Unmet challenges for rehabilitation after stroke in China. *Lancet* 2017, 390(10090):121-122.

**Table S1. Lifetime cost-effectiveness results for subgroups**

| **Subgroup** | **Lifetime Cost ($/￥)** | | | **QALYs** | | | **ICER**  **($/QALY)**  **(￥/QALY)** | **%<1 time of GDP/capita**  **per QALY** | **%<2 times of GDP/capital**  **per QALY** | **%<3 times of GDP/capita**  **per QALY** |
| --- | --- | --- | --- | --- | --- | --- | --- | --- | --- | --- |
|  | **Enalapril-folic acid** | **Enalapril** | **∆** | **Enalapril-folic acid** | **Enalapril** | **∆** |  |  |  |  |
| **Sex** | | | | | | | | | | |
| Male | $3,908.08  ￥25,207.12 | $2,578.25  ￥16,629.71 | $1,329.83  ￥8,577.40 | 10.61 | 10.52 | 0.09 | $14,091.71  ￥90,891.53 | 38.9% | 84.6% | 92.0% |
| Female | $4,180.21  ￥26,962.35 | $2,288.73  ￥14,762.31 | $1,891.48  ￥12,200.05 | 11.42 | 11.39 | 0.03 | $65,634.34  ￥423,341.49 | 0 | 1.5% | 13.0% |
| **Age, yrs** | | | | | | | | | | |
| <55 | $4,104.49  ￥26,473.96 | $1,903.12  ￥12,275.12 | $2,201.38  ￥14,198.90 | 12.98 | 12.94 | 0.04 | $64,893.23  ￥418,561.33 | 0 | 0.9% | 11.0% |
| ≥55 - <65 | $4,099.68  ￥26,442.94 | $2,608.56  ￥16,825.21 | $1,491.11  ￥9,617.66 | 11.22 | 11.12 | 0.09 | $15,956.14  ￥102,917.10 | 27.0% | 79.5% | 91.1% |
| ≥65 | $3,869.06  ￥26,442.94 | $2,499.96  ￥16,825.21 | $1,369.10  ￥9,617.66 | 9.06 | 9.04 | 0.02 | $56,003.24  ￥102,917.10 | 0 | 6.5% | 25.7% |
| **Smoking status** | | | | | | | | | | |
| Never | $3,909.34  ￥25,215.24 | $2,243.83  ￥14,472.70 | $1,665.51  ￥10,742.54 | 11.02 | 10.96 | 0.06 | $29,445.88  ￥189,925.93 | 0.7% | 37.5% | 68.8% |
| Current | $4,450.06  ￥28,702.89 | $2,807.46  ￥18,108.12 | $1,642.60  ￥10,594.77 | 11.06 | 10.99 | 0.07 | $24,746.65  ￥159,615.89 | 3.2% | 51.3% | 77.8% |
| Former | $4,142.53  ￥26,719.32 | $2,631.25  ￥16,971.56 | $1,511.29  ￥9,747.82 | 10.90 | 10.81 | 0.08 | $18,146.57  ￥117,045.38 | 18.6% | 74.5% | 87.1% |
| **Self-reported diabetes** | | | | | | | | | | |
| No | $4,023.52  ￥25,951.70 | $2,353.34  ￥15,179.04 | $1,670.18  ￥10,772.66 | 11.15 | 11.09 | 0.06 | $29,025.15  ￥187,212.22 | 1.0% | 37.1% | 67.4% |
| Yes | $4,909.14  ￥31,663.95 | $3,760.24  ￥24,253.55 | $1,148.89  ￥7,410.34 | 11.09 | 10.93 | 0.16 | $7,166.21  ￥46,222.05 | 86.5% | 96.2% | 97.7% |
| ***MTHFR* C677T genotype** | | | | | | | | | | |
| *MTHFR* CC | $3,863.26  ￥24,918.03 | $2,415.62  ￥15,580.75 | $1,447.65  ￥9,337.34 | 11.12 | 11.01 | 0.10 | $13,856.08  ￥89,371.72 | 39.2% | 86.6% | 93.2% |
| *MTHFR* CT | $4,065.94  ￥26,225.31 | $2,198.81  ￥14,182.32 | $1,867.13  ￥12,042.99 | 11.06 | 11.04 | 0.02 | $104,130.06  ￥671,638.89 | 0 | 0.1% | 2.6% |
| *MTHFR* TT | $4,232.09  ￥27,296.98 | $2,805.27  ￥18,093.99 | $1,426.82  ￥9,202.99 | 11.01 | 10.91 | 0.10 | $13,982.14  ￥90,184.80 | 40.5% | 84.8% | 92.1% |
| **Baseline SBP, mmHg** | | | | | | | | | | |
| <160 | $3,666.17  ￥23,646.80 | $1,704.23  ￥10,992.28 | $1,961.93  ￥12,654.45 | 11.18 | 11.17 | 0.01 | $391,445.20  ￥2,524,821.54 | 0 | 0 | 0 |
| ≥160 - <180 | $3,988.06  ￥25,722.99 | $2,258.30  ￥14,566.04 | $1,729.76  ￥11,156.95 | 11.05 | 11.00 | 0.05 | $36,225.97  ￥233,657.51 | 0.3% | 20.0% | 50.6% |
| ≥180 | $4,865.97  ￥31,385.51 | $3,795.50  ￥24,480.98 | $1,070.47  ￥6,904.53 | 10.93 | 10.76 | 0.16 | $6,502.14  ￥41,938.80 | 88.8% | 95.8% | 97.0% |
| **Total cholesterol, mmol/L** | | | | | | | | | | |
| <5.2 | $3,895.54  ￥25,126.23 | $1,934.81  ￥12,479.52 | $1,960.73  ￥12,646.71 | 11.00 | 11.00 | 0 | Enalapril dominat | 0 | 0 | 0 |
| ≥5.2 - <6.2 | $3,838.80  ￥24,760.26 | $2,345.51  ￥15,128.54 | $1,493.29  ￥9,631.72 | 11.11 | 11.02 | 0.09 | $16,355.43  ￥105,492.52 | 23.0% | 80.9% | 92.6% |
| ≥6.2 | $4,190.31  ￥27,027.50 | $2,922.60  ￥18,850.77 | $1,267.71  ￥8,176.73 | 10.99 | 10.86 | 0.13 | $10,018.65  ￥64,620.29 | 70.7% | 90.7% | 94.0% |
| **HDL-C, mmol/L** | | | | | | | | | | |
| <1.2 | $4,334.50  ￥27,957.53 | $2,475.96  ￥15,969.94 | $1,858.54  ￥11,987.58 | 11.07 | 11.05 | 0.02 | $85,060.49  ￥548,640.16 | 0 | 0.2% | 5.2% |
| 1.2 - 2.0 | $4,218.15  ￥27,207.07 | $2,639.53  ￥17,204.97 | $1,578.62  ￥10,182.10 | 11.11 | 11.03 | 0.08 | $19,708.32  ￥127,118.66 | 11.6% | 67.7% | 85.2% |
| ≥2.0 | $4,111.88  ￥26,521.63 | $2,622.42  ￥16,914.61 | $1,489.46  ￥9,607.02 | 11.18 | 11.07 | 0.10 | $14,503.64  ￥93,548.48 | 35.1% | 84.7% | 91.9% |
| **Folate, ng/mL** | | | | | | | | | | |
| <5.6 | $3,993.37  ￥25,757.24 | $2,753.08  ￥17,757.37 | $1,240.29  ￥7,999.87 | 11.13 | 10.98 | 0.15 | $8,430.69  ￥54,377.95 | 79.9% | 93.7% | 96.7% |
| ≥5.6-<10.5 | $4,138.41  ￥26,692.74 | $2,357.70  ￥15,207.17 | $1,780.71  ￥11,485.58 | 11.00 | 10.96 | 0.03 | $55,213.06  ￥356,124.24 | 0.1% | 4.0% | 21.8% |
| ≥10.5 | $4,108.41  ￥26,499.24 | $2,270.76  ￥14,646.40 | $1,837.64  ￥11,852.78 | 11.07 | 11.05 | 0.02 | $81,051.25  ￥522,780.56 | 0 | 0.3% | 6.5% |
| **Homocysteine, μmol/L** | | | | | | | | | | |
| ≤10 | $3,953.55  ￥25,500.40 | $2,065.80  ￥13,324.41 | $1,887.75  ￥12,175.99 | 11.05 | 11.04 | 0.02 | $117,532.71  ￥758,085.98 | 0 | 0.1% | 1.3% |
| 10 - 15 | $4,052.42  ￥26,138.11 | $2,424.84  ￥15,640.22 | $1,627.88  ￥10,497.89 | 11.12 | 11.06 | 0.06 | $25,129.99  ￥162,088.44 | 2.2% | 48.6% | 75.7% |
| >15 | $4,158.05  ￥26,819.42 | $2,469.38  ￥17,088.50 | $1,508.67  ￥9,730.92 | 11.05 | 10.96 | 0.09 | $16,740.92  ￥107,978.93 | 22.6% | 78.3% | 89.4% |

**Table S2. Baseline characteristics of the study participants**

| **Characteristics** | **Enalapril-folic acid group**  **(n=10348)** | **Enalapril group (n=10354)** | **P value** |
| --- | --- | --- | --- |
| **Sociodemographic characteristics** | | | |
| Age, yrs (SD) | 60.0 (7.5) | 60.0 (7.6) | 0.543 |
| Male, n (%) | 4245 (41.0) | 4252 (41.1) | 0.949 |
| Body mass index, kg/m2 (SD) | 25.0 (3.7) | 24.9 (3.7) | 0.467 |
| **Clinical characteristics** | | | |
| MTHFR C677T genotype, n (%) |  |  | 0.973 |
| CC | 2821 (27.3) | 2831 (27.3) |  |
| CT | 5095 (49.2) | 5081 (49.1) |  |
| TT | 2432 (23.5) | 2442 (23.6) |  |
| Smoking status, n (%) |  |  | 0.359 |
| Never | 7119 (68.8) | 7135 (68.9) |  |
| Former | 761 (7.4) | 809 (7.8) |  |
| Current | 2461 (23.8) | 2408 (23.3) |  |
| Self-reported diabetes, n (%) | 317 (3.1) | 335 (3.2) | 0.474 |
| Baseline SBP, mm Hg (SD) | 166.8 (20.4) | 166.9 (20.4) | 0.713 |
| Mean SBP during treatment, mm Hg (SD) | 139.7 (11.1) | 139.8 (11.3) | 0.637 |
| Total cholesterol, mg/dL (SD) | 213.6 (46.0) | 213.2 (45.8) | 0.613 |
| HDL-C, mg/dL (SD) | 52.0 (14.0) | 51.8 (13.9) | 0.222 |
| Fasting glucose, mg/dL (SD) | 104.5 (30.6) | 104.5 (30.6) | 0.274 |
| Baseline folate, ng/mL (SD) | 8.5 (4.0) | 8.5 (4.0) | 0.830 |
| Homocysteine, µmol/L (IQR) | 12.5 (10.5 – 15.5) | 12.5 (10.5 – 15.5) | 0.829 |
| **Medication use, n (%)** | | | |
| Angiotensin-converting enzyme inhibitors | 938 (9.1) | 955 (9.2) | 0.220 |
| Angiotensin II receptor blockers | 10 (0.1) | 8 (0.1) | 0.490 |
| Calcium channel blockers | 1034 (10.0) | 1035 (10.0) | 0.311 |
| Diuretics | 218 (2.1) | 217 (2.1) | 0.467 |
| β-Blockers | 84 (0.8) | 91 (0.9) | 0.766 |
| Lipid-lowering drugs | 81 (0.8) | 85 (0.8) | 0.467 |
| Glucose-lowering drugs | 166 (1.6) | 151 (1.5) | 0.056 |
| Antiplatelet drugs | 285 (2.8) | 322 (3.1) | 0.159 |

MTHFR=methylenetetrahydrofolate reductase; HDL-C=high-density lipoprotein cholesterol; SBP=systolic blood pressure.

**Table S3. Unit prices of drugs**

| **Drug** | **Specification** | **Market share (%)** | | **Price ($)** | | |
| --- | --- | --- | --- | --- | --- | --- |
|  |  | **Unadjusted** | **Adjusted** | **Low** | **Value** | **Up** |
| Enalapril | 10mg | **/** | **/** | 0.03 | 0.09 | 0.22 |
| **Angiotensin converting enzyme inhibitors (ACEI)** | | | | | | |
| Perindopril tert-butylamin tablets | 4mg | 35.5 | 50.2 | 0.11 | 0.12 | 0.43 |
| Benazepril hydrochloride tablets | 10mg | 25.0 | 35.4 | 0.11 | 0.15 | 0.40 |
| Fosinopril sodium tablets | 10mg | 10.2 | 14.4 | 0.13 | 0.13 | 0.13 |
| Total weighted price |  |  |  | 0.11 | 0.13 | 0.38 |
| **Angiotensin II receptor blockers** | | | | | | |
| Valsartan capsules | 80mg | 18.7 | 35.8 | 0.03 | 0.04 | 0.54 |
| Losartan tablets | 50mg | 13.4 | 25.7 | 0.16 | 0.16 | 0.70 |
| Irbesartan tablets | 150mg | 12.7 | 24.3 | 0.05 | 0.05 | 0.49 |
| Telmisartan tablets | 40mg | 7.4 | 14.2 | 0.07 | 0.09 | 0.32 |
| Total weighted price |  |  |  | 0.07 | 0.08 | 0.54 |
| **Calcium channel blockers (CCB)** | | | | | | |
| Amlodipine Besylate tablets | 5mg | 31.3 | 38.0 | 0.01 | 0.02 | 0.53 |
| Nifedipine controlled-release tablets | 30mg | 24.5 | 29.8 | 0.36 | 0.53 | 0.54 |
| Levamlodipine Besylate tablets | 2.5mg | 18.1 | 22.0 | 0.08 | 0.26 | 0.30 |
| Felodipine sustained-release tablets | 5mg | 8.4 | 10.2 | 0.14 | 0.24 | 0.43 |
| Total weighted price |  |  |  | 0.14 | 0.25 | 0.47 |
| **Diuretics** | | | | | | |
| Torasemide tablets | 10mg | 75.1 | 82.6 | 0.32 | 0.33 | 0.33 |
| Spironolactone tablets | 20mg | 9.5 | 10.5 | 0.02 | 0.03 | 0.05 |
| Indapamide tablets | 2.5mg | 6.3 | 6.9 | 0.01 | 0.25 | 0.32 |
| Total weighted price |  |  |  | 0.27 | 0.29 | 0.30 |
| **β-Blockers** | | | | | | |
| Metoprolol tartrate tablets | 25mg | 45.7 | 64.7 | 0.01 | 0.02 | 0.05 |
| Bisoprolol fumarate tablets | 5mg | 24.9 | 35.3 | 0.05 | 0.06 | 0.06 |
| Total weighted price |  |  |  | 0.02 | 0.03 | 0.05 |
| **Lipid-lowering drugs** | | | | | | |
| Atorvastatin calcium tablets | 10mg | 52.2 | 60.7 | 0.02 | 0.05 | 0.56 |
| Rosuvastatin calcium tablets | 10mg | 22.2 | 25.8 | 0.03 | 0.12 | 0.86 |
| Simvastatin tablets | 20mg | 8.3 | 9.7 | 0.02 | 0.03 | 0.34 |
| Pitavastatin calcium tablets | 2mg | 3.3 | 3.8 | 0.06 | 0.16 | 0.79 |
| Total weighted price |  |  |  | 0.03 | 0.08 | 0.65 |
| **Glucose-lowering drugs** | | | | | | |
| Acarbose tablets | 50mg | 37.0 | 45.0 | 0.03 | 0.03 | 0.23 |
| Metformin hydrochloride tablets | 0.85g | 19.0 | 23.0 | 0.01 | 0.02 | 0.15 |
| Repaglinide tablets | 1mg | 9.0 | 11.0 | 0.04 | 0.05 | 0.21 |
| Gliclazide | 30mg | 7.0 | 9.0 | 0.09 | 0.13 | 0.22 |
| Glimepiride tablets | 2mg | 10.0 | 12.0 | 0.01 | 0.03 | 0.51 |
| Total weighted price |  |  |  | 0.03 | 0.04 | 0.24 |
| **Antiplatelet drugs** |  |  |  |  |  |  |
| Aspirin enteric-coated tablets | 0.1g | / | / | 0.05 | 0.05 | 0.08 |

**Table S4. Costs of each treatment procedure**

| **Treatment procedure** | **Unit Price ($)** | | | **Counts/patient** |
| --- | --- | --- | --- | --- |
|  | **Low** | **Baseline** | **Up** |  |
| **Rehabilitation training** | | | | |
| Motortherapy | 2.11 | 2.37 | 4.65 | 5 times a week |
| Cognitive dysfunction therapy | 2.33 | 5.58 | 6.20 | Once per week |
| Speech training | 2.33 | 6.98 | 7.75 | Once per week |
| Swallowing dysfunction therapy | 2.33 | 2.79 | 3.10 | Once per week |
| Recreational therapy | 0.74 | 0.84 | 3.10 | 5 times a week |
| Occupational therapy (include activities of daily living) | 2.33 | 2.79 | 3.10 | 5 times a week |
| Traditional Chinese massage | 2.48 | 2.79 | 5.43 | Once per week |
| Acupuncture | 1.55 | 2.09 | 2.33 | Once per week |
| **Total cost per month** | 147.60 | 200.93 | 316.28 |  |
| **Rehabilitation checking** | | | | |
| Family sickbed service | 1.55 | 2.33 | 2.64 | Once per month |
| Rehabilitation evaluation | 2.33 | 5.58 | 6.20 | Once per month |
| **Total cost per month** | 3.88 | 7.91 | 8.84 |  |
| **Home care cost per month** | 372.09 | 465.12 | 558.14 |  |

**Table S5. Hazard rations and parameters for scale and shape of subgroup analysis**

| **Variable** | **λ** | **γ** | **HR (95% CI)** |
| --- | --- | --- | --- |
| **Sex** |  |  |  |
| Male | 0.0128 | 0.8189 | 0.68 (0.54 - 0.86) |
| Female | 0.0074 | 0.9262 | 0.90 (0.73 - 1.12) |
| **Age, yrs** |  |  |  |
| <55 | 0.0064 | 0.8009 | 0.87 (0.60 - 1.26) |
| 55 -65 | 0.0093 | 0.9219 | 0.70 (0.55 - 0.89) |
| ≥65 | 0.0135 | 0.8396 | 0.86 (0.67 - 1.10) |
| **Smoking status** |  |  |  |
| Never | 0.0080 | 0.9171 | 0.79 (0.64 - 0.96) |
| Current | 0.0126 | 0.8139 | 0.80 (0.60 - 1.06) |
| Former | 0.0145 | 0.7787 | 0.76 (0.46 - 1.26) |
| **Self-reported diabetes** |  |  |  |
| No | 0.0096 | 0.8636 | 0.80 (0.68 - 0.94) |
| Yes | 0.0119 | 0.9889 | 0.65 (0.30 - 1.43) |
| ***MTHFR* C677T genotype** |  |  |  |
| *MTHFR* CC | 0.0097 | 0.8705 | 0.65 (0.48 - 0.89) |
| *MTHFR* CT | 0.0090 | 0.8421 | 0.93 (0.74 - 1.16) |
| *MTHFR* TT | 0.0110 | 0.9120 | 0.72 (0.53 - 0.97) |
| **Baseline SBP, mmHg** |  |  |  |
| <160 | 0.0050 | 0.8956 | 0.97 (0.70 - 1.34) |
| 160 - 180 | 0.0091 | 0.8461 | 0.82 (0.63 - 1.07) |
| ≥180 | 0.0179 | 0.8861 | 0.69 (0.54 - 0.88) |
| **Total cholesterol, mmol/L** |  |  |  |
| <5.2 | 0.0076 | 0.8493 | 1.00 (0.77 - 1.29) |
| 5.2 - 6.2 | 0.0106 | 0.8448 | 0.70 (0.53 - 0.92) |
| ≥6.2 | 0.0119 | 0.9196 | 0.68 (0.51 - 0.90) |
| **HDL-C, mmol/L** |  |  |  |
| <1.2 | 0.0091 | 0.8644 | 0.92 (0.71 - 1.19) |
| 1.2 - 2.0 | 0.0100 | 0.8669 | 0.73 (0.59 - 0.90) |
| ≥2.0 | 0.0091 | 0.9062 | 0.67 (0.33 - 1.38) |
| **Folate, ng/mL** |  |  |  |
| <5.6 | 0.0152 | 0.7796 | 0.61 (0.46 - 0.81) |
| 5.6 - 10.5 | 0.0088 | 0.8917 | 0.88 (0.70 - 1.09) |
| ≥10.5 | 0.0055 | 1.0419 | 0.91 (0.64 - 1.29) |
| **Homocysteine, μmol/L** |  |  |  |
| ≤10 | 0.0069 | 0.9024 | 0.93 (0.63 - 1.35) |
| 10 - 15 | 0.0085 | 0.9243 | 0.78 (0.63 - 0.98) |
| >15 | 0.0138 | 0.7877 | 0.74 (0.57 - 0.97) |
