# Supplementary material for: Cost-effectiveness of folic acid therapy for primary prevention of stroke in patients with hypertension
Source: BMC Med. 2022 Oct 25;20:407. doi: 10.1186/s12916-022-02601-z (PMC9594871; doi:10.1186/s12916-022-02601-z)
Supplement: Supplementary file 2 — Additional file 2: Figure S1. Schematic structure of the microsimulation model. Figure S2. Model of internal validation. Internal validation of our microsimulation model shows agreement with the 4.5-year period of in-trial data across first stroke rates. [file 12916_2022_2601_MOESM2_ESM.docx]

**Cost-Effectiveness of Folic Acid** **Therapy for Primary Prevention of Stroke in Patients with Hypertension**

**Additional file 2**

**
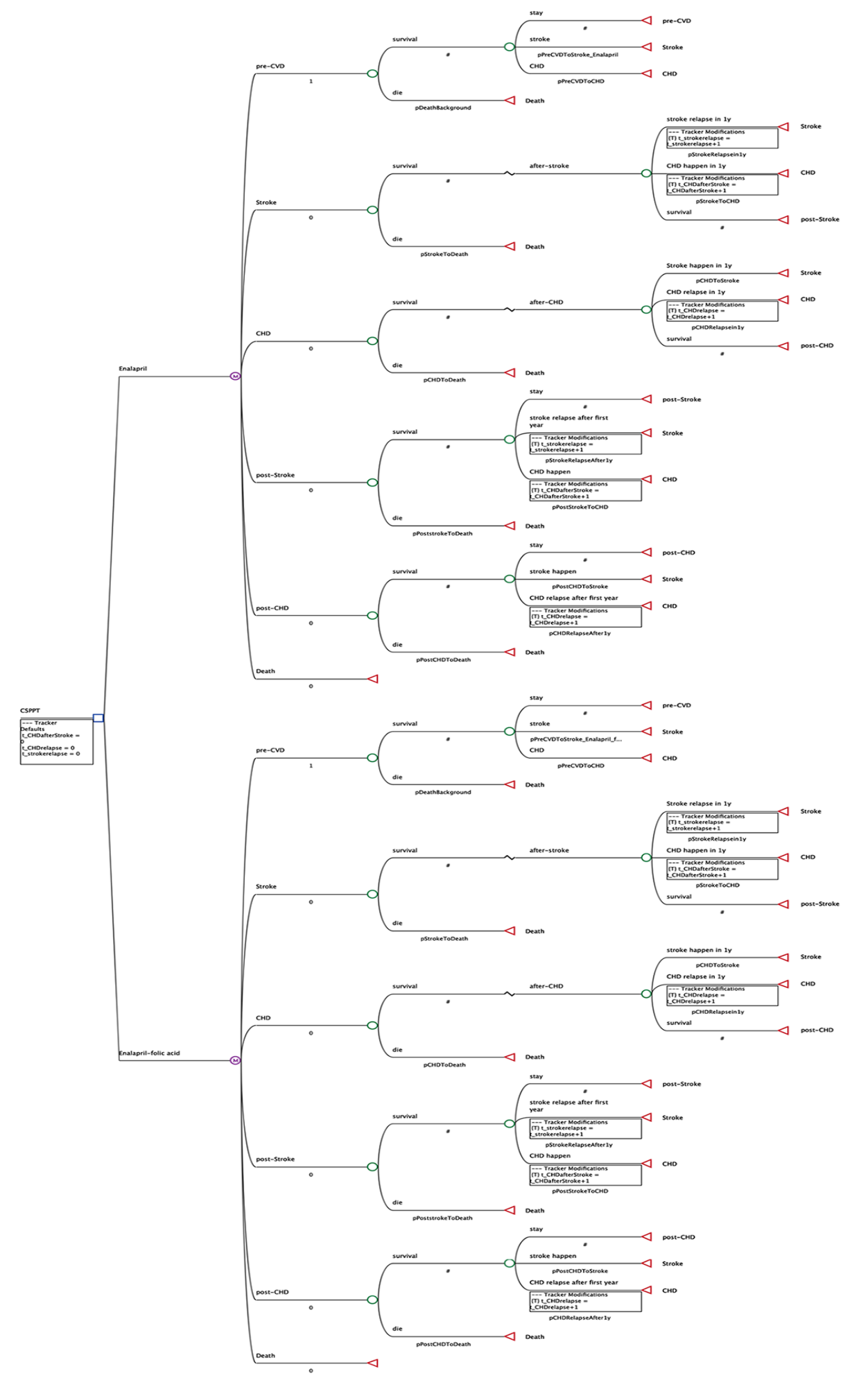
**

**Figure S1. Schematic structure of the microsimulation model.**


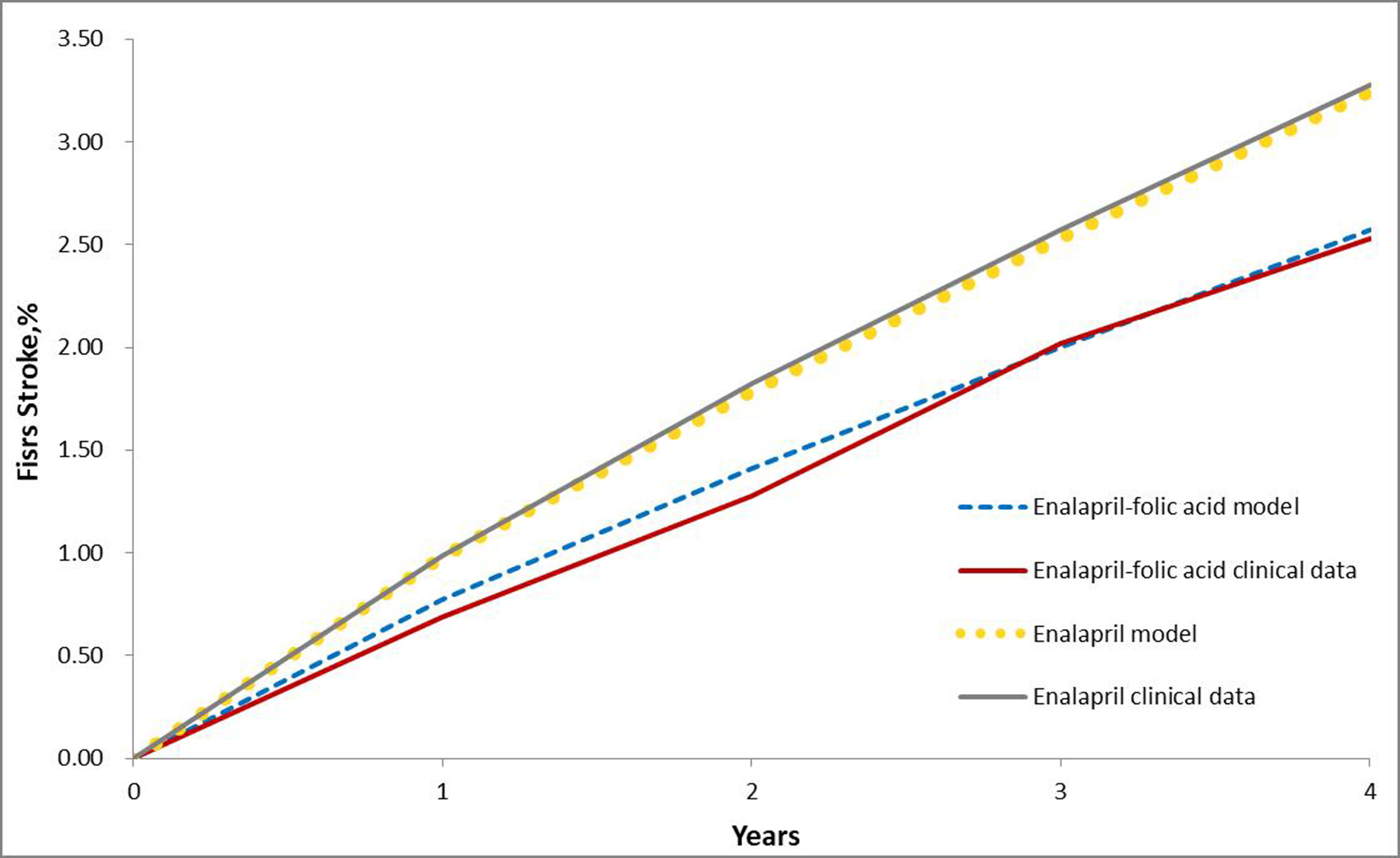


**Figure S2. Model of internal validation.** Internal validation of our microsimulation model shows agreement with the 4.5-year period of in-trial data across first stroke rates
